# Remember Absent Friends

**Published:** 1915-12-18

**Authors:** 


### REMEMBER ABSENT FRIENDS.

Another Christmas is upon us, and more than ever of our gallant defenders will spend it in the trenches. Not only is our Army holding a far longer line in Flanders and Artois than it did last year—twice or three times as long, indeed—but our commitments in Macedonia, in Gallipoli, in Mesopotamia, in East Africa, and in the Cameroons are vastly in excess of anything even contemplated a year ago. And our incomparable Navy's vigil in the North Sea—did we ever really allow ourselves to hear it called the German Ocean?—is as incessant and as inexorable as ever, to say nothing of the extended fields of strife in the Mediterranean, in the Baltic, and in the Sea of Marmora. At this season it is right and natural that the thoughts of all who are in England should turn to our brave lads, ay, and lasses too, in the theatres of war, with earnest wishes for their safety and their success in all their undertakings. With the personal interest which everyone takes in their relatives, friends, and neighbours who are upholding Britain's honour, and the impersonal pride which every normal individual feels in his country's sailors and soldiers, there is inevitably one other sentiment that the coming of Christmas is bound to suggest: the longing, that is, for peace and for the end of this criminal carnage that is deluging Europe with blood and fire. That consummation, devoutly though it is desired and prayed for, looks still to be a long way off: for peace to be worthy of the name must not be only peace with honour, but peace with the assurance that it will be lasting, and that tyrants who may hereafter wax ambitious will think twice before they tear up treaties and order those calculated atrocities which have indelibly and for ever stained the German Army. Whether the next ensuing festival of the birth of the Prince of Peace will see such a peace established on earth no man can say: this Christmastide, at any rate, seems far enough away from it.

To the men at the Front the desire for peace is, in most cases, as ardent as it is among those at home; but they too are well aware that the only peace worth having is one which will prevent the recurrence of the present evils for some generations. Their thoughts will turn homewards, no doubt, just as ours turn to them abroad. Huddling in icy dug-outs or broiling in the Tropics, wading in the slush of the trenches, or packed like sardines in "reserve billets," the exiles may be tempted for a moment to wonder whether their hardships, their sacrifices, and their heroism are duly understood at home. It is true to some extent that civilian opinion at home finds it hard to get the correct angle of vision for much that takes place at the Front; but this much can confidently be asserted, that those at home send the warmest of Christmas greetings to those abroad, in whatever theatre of war they are serving. Our gratitude to them is even intensified at this season, because we realise more the comforts and homes which they are compelled to do without. We shall think of them, just as we know that they will be thinking of us; and we wish them to know and realise that their labours are appreciated in the Old Country.

These considerations apply just as much to the institutional world as to the rest of the community. Pre-occupied with the daily round, carried through in many institutions at ever-increasing pressure owing to the demands of the Army for doctors, nurses, and able-bodied males of all kinds; and with the preparations for such modified Yuletide festivities as are feasible in the circumstances, it might be supposed that institutional workers, whose tasks have to be done on Sundays as well as week-days, Bank holidays as well as other days, would have less time than most folks for thoughts of absent friends on duty against the enemy. We are confident that this will not be found to be the case, and that nowhere will anxious hearts and kindly wishes for our heroes abroad be more universal than in the institutional world.

Most of the absent comrades and fellow-workers from our institutions are serving in some capacity or another in the system for the relief of pain and injury, in the Royal Army Medical Corps, Queen Alexandra's Imperial Military Nursing Service, the Red Cross, or some kindred employ; though a fair number of institutional porters, carpenters, and similar employees are in the combatant branches. Some of them are decently housed and carry on their tasks in comparative comfort; many more have at least the moderate shelter of tents or "billets" of some kind; probably only a minority will actually spend their Christmas Day in the trenches, or under fire. One way or another they will manage to keep their Christmas: last year a temporary and strictly unofficial truce was observed, but we doubt whether this year our soldiers will feel inclined to repeat the experiment, and probably the Germans, on their side, will be discouraged by orders from their Higher Command, which was reported to be much displeased with the occurrences on Christmas Day last year. On our side, each and all are, we feel sure, doing their duty as it lies before them, with all their might; and for this they are entitled to the grateful thanks of all of us at home, and to a special remembrance of their labours at this particular season.

